# Sex Differences in the Metabolic Cost of a Military Load Carriage Task: A Field Based Study

**DOI:** 10.3390/sports13120442

**Published:** 2025-12-09

**Authors:** Ben Schram, Jacques Rosseau, Elisa F. D. Canetti, Robin Orr

**Affiliations:** 1 Tactical Research Unit, Bond University, Robina, QLD 4226, Australia; ecanetti@bond.edu.au (E.F.D.C.); rorr@bond.edu.au (R.O.); 2 Human Performance Cell, Joint Support Group, New Zealand Army, Waiouru 4825, New Zealand; jacques.rosseau@nzdf.mil.nz

**Keywords:** pack march, aerobic fitness, occupational tasks, army

## Abstract

Occupational demands, such as load carriage in tactical professions, do not discriminate based on sex. The aim of this study was to explore the differences in metabolic cost of a loaded pack march between the sexes in both absolute and relative terms. Twelve Army personnel (six males and six females) volunteered to complete three identical load carriage marches (5 km at 5.5 km/h, carrying 30 kg), across flat (on road) and undulating (gravelled path) terrain as part of a larger equipment trial. Heart rate (HR) response (HR average and maximum) was monitored with a Polar Team Pro unit and oxygen consumption with VO Master Pro (VO_2_ average and maximum) with the level of significance set at 0.05. There were no significant differences in age, years of experience, absolute loads carried, or completion time for each of the three events. Male soldiers were significantly taller (182.3 ± 6.2 cm vs. 167.4 ± 6.9 cm), heavier (88.2 ± 8.7 kg vs. 70.9 ± 10.6 kg), carried significantly less relative load (34.3 ± 3.4% vs. 43.2 ± 7.5%), and had significantly greater predicted VO_2max_ (56.7 ± 6.1 mL/kg/min vs. 45.0 ± 2.9 mL/kg/min). A linear mixed model identified a significant main effect of sex on both average HR (β = −1.10) and peak HR (β = −1.27), and on average VO_2_ (β = −0.68), but not peak VO_2_. While the study was not powered to detect sex differences, the large effect sizes observed suggest meaningful physiological differences warranting further investigation. Female soldiers faced significantly greater metabolic costs when carrying the same loads and moving at the same speed and across the same terrain as their male counterparts. Adequate recovery and pacing strategies should be considered for these events, especially during training.

## 1. Introduction

Load carriage is a fundamental requirement in military operations, with historical evidence highlighting its significant impact on force wide mobility, lethality and cognitive performance [1,2]. Operational loads exceeding 45 kg are increasingly common [1,3,4] and while designed to improve survivability, such loads have been shown to decrease mobility [5,6] impair marksmanship [7,8] reduce grenade throw distance [9] and increase vulnerability during fire and movement drills [10]. In addition to these decrements, attention-to-task is impacted, most notably to visual stimuli [11,12,13], critical for threat identification.

Although load carriage requirements are dictated by mission demands, they are applied uniformly across personnel, regardless of sex, age, or body size. With the removal of gender-based role restrictions and the growing representation of females in direct combat roles [14], understanding sex-specific responses to load carriage has become increasingly important. Female soldiers are generally shorter and lighter than male soldiers [15], yet they carry similar absolute loads, which can equate to up to 50% of their bodyweight [16]. While progressive load carriage training is standard in most militaries [4], training loads (~10–20 kg) are often substantially lighter than operational loads (25–45 kg) [17], potentially underpreparing personnel for real world demands.

Load carriage is associated with a range of musculoskeletal injuries [1,18] and emerging evidence highlights sex-based differences in these injury profiles. Females are more prone to foot injuries, males experience higher rates of ankle injuries [19], and both groups report similar rates of back injuries. However the severity of injuries among females soldiers tend to be greater, likely reflecting their smaller stature [19]. Laboratory studies have shown that wearing combat gear significantly reduces cardiorespiratory capacity and time to exhaustion in both sexes, with females carrying relatively heavier loads despite similar absolute mass [20]. Biomechanically, females tend to walk with a higher cadence, longer stance phases and greater pelvis, hip and knee motion, alongside increased trunk lean and ground reaction forces [21,22]. External load has also been shown to increase lower limb gait variability in females, suggesting greater neuromuscular influences lower-limb gait variability in females during load carriage with greater neuromuscular adaptation demands under heavy loads [23]. Interestingly, females may experience less neuromuscular fatigue post load carriage, potentially due to differences in skeletal muscle physiology [24].

Given the operational burden of load carriage, and the disproportionate strain it may place on female soldiers, there remains a critical gap in field-based data examining sex-based physiological responses under operationally realistic loads and conditions. Sex differences in metabolic cost may stem from disparities in aerobic capacity, muscle fibre composition, and gait mechanics. Relative load interacts with absolute load and fitness, influencing physiological strain. Field-based studies are essential to capture terrain variability and operational realism absent in laboratory protocols. Most existing studies have been conducted in controlled laboratory settings, often using treadmill protocols or simulated gear configurations that may not fully capture the demand of real-world military tasks. This study addresses that gap by examining sex-based differences in the metabolic cost of a standardised military load carriage task conducted in a field setting, with a specific focus on both absolute (e.g., heart rate, oxygen consumption) and relative (e.g., %VO_2_ max) physiological demands.

## 2. Materials and Methods

A prospective cohort study was designed as part of a larger study which was designed to trial combat packs for Army personnel. Within this broader study, three different backpack variants were trialled over a one-week period, with a trial on Monday, Wednesday, and Friday with a rest period (Tuesday and Thursday) between subsequent trials. For this study, the differences in metabolic cost between male and female soldiers was investigated. Load weight was the independent variable and heart rate and VO_2_ were the dependent variables. While absolute energy cost reflects the task burden, relative metrics (e.g., %VO_2_ max) are critical for understanding individual strain, recovery needs, and injury risk.

The participants were 12 Army soldiers (♂ n = 6; ♀ n=6; mean age = 26.4 ± 5.6 years; mean years of service 6.7 ± 5.0 years; mean height = 174.8 ± 10.0 cm; mean weight = 79.5 ± 12.9 kg, load weight = 30 kg, relative weight = 38.8 ± 7.2% body weight, mean predicted VO_2_ = 50.8 ± 7.6 mL/kg/min). Participants represented six corps/trades (logistics, infantry, engineers, dental, intelligence, and officer cadets). with varied fitness and anthropometrics. This heterogeneity reflects real-world military populations but introduces variability that may influence interpretation. All personnel provided written informed consent to participant. Ethics approval for this study was provided from University Human Research Ethics Committee (BS02246 amendment 1). The research was carried out in accordance with the conditions of the Declaration of Helsinki, recommendations guiding physicians in biomedical research involving human subjects [25], and the Australian National Health and Medical Research Council [26]. 

Participants were randomised to one of the three military pack conditions which all met requirements for capacity, fabric, etc., per tender specifications for military use; however, these were not supplied to the researchers in order to avoid bias. There were no differences in structural characteristics or load distribution characteristics which could influence metabolic cost. Soldiers were allowed to self-pack their packs and adjust any systems associated with that pack according to preference, noting that, as in previous research of this nature [27], some packs were more adjustable than others. Following this allocation personnel provided basic demographic details (e.g., date of birth, sex, years of service, corps) before their height was measured (Seca, Hamburg, Germany), and they were weighed on a portable scale (UWE-AFM [300 kg]). Participants’ most recent 2.4 km run times (which comprised part of their bi-annual fitness assessment) were provided from military records and were converted into a predicted VO_2max_ using a validated regression equation by Berger 1990 which explains up to 92% of the variance with a standard error of 2.24–2.91 mL/kg/min [28]. Personnel were fitted with a Polar Team heart rate (HR) monitor (Polar Electro Oy, Kempele, Finland) and proceeded to pack their allocated pack with required equipment. Each soldier was provided with a blue rifle (weight 4.3 kg). Total load was measured (Camry Hanging Scale [100 kg]) and checked by the researchers to ensure they equated to 30 kg in total. Soldiers were allowed to self-pack their packs and adjust any systems associated with that pack according to preference, noting that, as in previous research of this nature [29], some packs were more adjustable than others. Due to equipment availability and operational constraints during field testing, six of the 12 personnel volunteered to wear a portable device (VO2MASTER, VO2 Master Health Sensors Inc., Vernon, B.C., Canada) used to measure oxygen consumption, utilisation, and uptake (VO_2_). There were no significant differences between wearers and non-wearers in terms of age, body mass, aerobic fitness, or load carriage experience. These devices were fitted after being flow calibrated with a 3 L syringe and calibrated for ambient air per the manufacturer’s instructions. Given the march was submaximal, the medium mouthpiece (ventilation range 15–180 L/min) for the VO2MASTER was used for all participants.

Personnel then moved outside and completed a 5 km walk at a pace of 5.5 km/h across flat and undulating terrain. Personnel were formed up as a group in a relaxed formation wearing their combat ensemble (30 kg total). The pace was set by a researcher at circa 5.5 km/h with participants allowed to set their own stride length and stride frequency. The 5 km course was around the Military Area beginning at an elevation of 825 m and consisted of 1.95 km on flat ground (mixture of bitumen and asphalt, 550 m incline to 1375 m (asphalt, 4.4° incline) followed by a reversal of direction (550 m downhill and 1.95 km flat ground) back to the starting point. Environmental conditions were monitored via a Kestrel wet bulb thermometer throughout testing and remained relatively constant (13.3–14.4 °C, 68.2–76.8% humidity). Objective outcome measures for this march were HR and VO_2_.

Heart Rate Measures: HR was measured via a Polar Team Pro chest strap (Polar Electro Oy, Kempele, Finland), with each participant fitted with a Polar Team HR monitor. These devices have been shown to be accurate [29] and valid in measuring heart rate in tactical populations [30]. Each participant had their chest strap individually fitted by a member of the research team and was checked to ensure viable data were being collected. HR data were collected at 10 Hz, with key outputs being average and maximum heart rates for each participant.

VO_2_ Measures: VO_2_ is a measure of the ability of the body to uptake, transport, and utilise oxygen and is a useful measure of aerobic fitness [31]. Not only has aerobic fitness been found to be the biggest predicter of load carriage performance [32], but it has also been associated with tactical task performance [33] and injury risk [34]. VO_2_ was measured (VO2MASTER, VO2 Master Health Sensors, Vernon, B.C., Canada) while participants completed their 5.5 km pack march. This device has been shown to be both valid and reliable in previous research [35,36]. Data were collected initially breath by breath but averaged over 30 s post hoc. The key variables were average and peak relative VO_2_ for the participants, reported in mL/kg/min.

### Statistical Analysis

Data were collected electronically on an excel spreadsheet and transferred into JASP (Version 0.l9.03). Key variables (i.e., age, years of experience, height, uniformed body weight, load, predicted VO_2_ and anthropometric measures) were descriptively analysed. Relative load (%) was the percentage of load carried in relation to the soldier’s body weight and was determined by dividing the load carried (30 kg) by the soldier’s body weight and multiplying the result by 100. Independent samples t-tests were performed to investigate differences in the above listed variables between sexes.

A linear mixed model (LMM) was used to analyse the effects of sex (male, female) and pack variant (A, B, C) on heart rate (HR) oxygen consumption (VO_2_), including both average and peak values. The model included sex, pack variant, and their interaction (sex*variant) as fixed effects, while participant ID was included as a random intercept to account for repeated measures within individuals.

Due to the small sample size, random slopes for sex and backpack variant were initially considered to account for potential variability in these effects across individuals. However, the inclusion of random slopes led to convergence warnings and inflated standard errors due to the small samples size and limited repeated measures per participant. These estimations issues indicated overparameterization relative to the available data, which can produce unreliable estimates. Therefore, the final model retained random intercepts only which is consistent with best practice for small-sample mixed models where random slops cannot be reliably estimated. Satterthwaite’s approximation [37,38] was used to estimate degrees of freedom for fixed effects, and Type III sum of squares was applied to assess statistical significance. The model was fitted using restricted maximum likelihood (REML), and results were reported as fixed effect estimates (Estimate (B), SE, t, and *p*-values). To facilitate effect size interpretation in the LMMs, standardised regression coefficients (β*) were computed post hoc following the approach outlined by Snijders and Bosker [39]. Standardised coefficients (*β*) were derived by adjusting the unstandardized fixed-effect estimates using the formula: *β* = B*SD_x_/SD_y_, where *B* is the unstandardized regression coefficient from the LMM output, SD_x_ is the standard deviation of the predictor variable, and SD_Y_ is the standard deviation of the outcome variables (e.g., average HR, peak HR, average VO_2_, peak VO_2_), obtained from descriptive statistics. For binary categorical predictors (e.g., sex: male = 0, female = 1), standardisation followed the recommendations of Gelman [40], by dividing 2 × SD which for a binary variable coded 0/1 equals 0.5. This approach places binary predictors on a comparable scale to continuous predictors (approximately one unit change corresponds to a two-standard deviation shift), improving interpretability of standardised coefficients in mixed models. The level of significance for statistical tests was set a priori at 0.05.

## 3. Results

Descriptive statistics for the soldiers’ demographics are shown in Table 1. There were no differences between sexes in age or years of service. The male soldiers were, on average, significantly taller (*t*(10) = −3.938, *p* = 0.001), heavier (*t*(10) = −3.091, *p* = 0.006), carried less relative load (*t*(10) = −2.265, *p* = 0.012) with a higher level of predicted aerobic fitness (*t*(10) = −4.272, *p* = 0.001) than the female soldiers.

**Figure 1:**
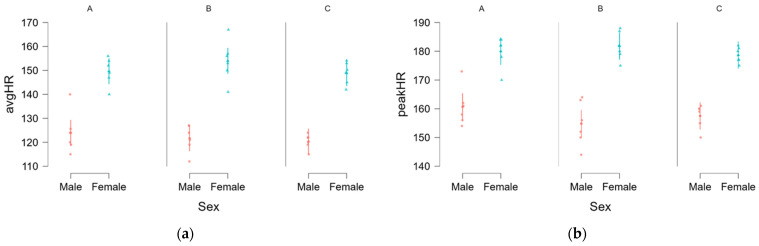
Sex differences amongst worn packs for average (**a**) and peak HR (**b**). A, B, C = Variant.

Heart rate: A linear mixed model was conducted to assess the effects of sex and variant on average and peak heart rate (HR), with participant included as a random effect. The results indicated a significant main effect of sex on average HR (*F*(1, 10.00) = 107.930, *p* < 0.001) with females exhibiting significantly higher average HR values (150.9 ± 6.8 bpm) than males (122.0 ± 6.1 bpm, Figure 1a). Similarly, a significant main effect of sex was also observed for peak heart rate (*F*(1, 10.00) = 72.188, *p* < 0.001) again showing that females had higher peak HR values (180.1 ± 4.6 bpm) than males (157.7 ± 6.5 bpm), Figure 1b).

The fixed-effects estimates further confirmed that sex was a significant predictor of both average HR (*B* = −28.872, SE = 1.390, *t*(10.00) = −10.389, *p* < 0.001, *β* = −1.099) and peak HR (*B* = −22.583, SE = 1.324, *t*(10.00) = −8.496, *p* < 0.001, *β* = −1.267) (Table 2), indicating that males consistently displayed lower HR values than females. However, no significant main effect of variant was found for either average HR or peak HR, suggesting that heart rate did not significantly differ between the three pack variants. Similarly, the sex*variant interaction was not significant for average heart rate HR, indicating that the sex difference in average HR was consistent across variants. For peak HR, the sex*variant interaction approached significance, with fixed effects estimates revealing a significant sex difference whilst carrying variant C, suggesting that males exhibited a notably lower peak HR in this condition compared to females. Estimated marginal means for average HR and peak HR are presented in Supplementary Table S1. Across all conditions, females exhibited higher heart rate values than males, though the interaction effects suggest some variability in peak HR differences depending on the variant.

**Figure 2:**
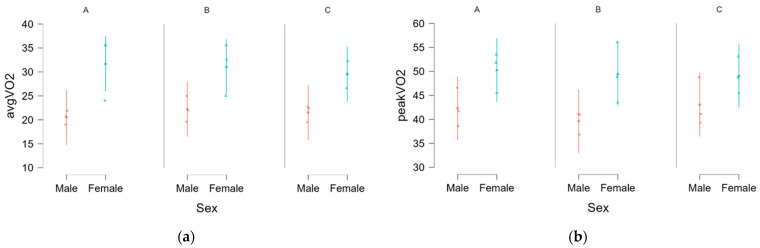
Sex differences amongst worn packs for average (**a**) and peak VO_2_ (**b**). A, B, C = Variant.

VO_2_: A linear mixed model was conducted to assess the effects of sex and variant on average and peak VO_2_, with participant included as a random effect. The results indicated a significant main effect of sex on average VO_2_, *F*(1, 4.00) = 9.860, *p* = 0.035, with females reaching significantly higher values of average VO_2_ (Figure 2a); however, this effect was not observed for peak VO_2_, *F*(1, 4) = 5.344, *p* = 0.082), (Figure 2b).

A summary of fixed effects estimates for both average and peak VO_2_ is presented in Table 3. The fixed effects estimate demonstrate a significant effect of sex for average VO_2_ (*B* = −4.678, SE = 1.49, *t*(4.00) = −3.14, *p* < 0.035, *β* = −0.682), with females exhibiting higher values than males, but, despite the same trend, this was not significant for peak VO_2_ (*B* = −3.972, SE = 1.718, *t*(4.00) = −2.312, *p* < 0.082, *β* = −0.598). No significant main effects of pack variant or interactions were found for either outcome measure. Estimated marginal means for average and peak VO_2_ are presented in Supplementary Table S2. Across all conditions, females exhibited higher VO_2_ values than males, though these differences were only statistically significant for average VO_2_.

## 4. Discussion

The aim of this study was to determine the differences in the metabolic cost of a military load carriage task between male and female soldiers. When carrying 30 kg of load over a 5 km route at 5.5 km/h female soldiers have a significantly greater metabolic demand as measured by HR and VO_2_ than male soldiers. There are few field-based studies of enlisted soldiers carrying operational loads to draw direct comparison to the results of this study.

A recent systematic review by Hudson et al. [41] found that both absolute oxygen uptake and minute ventilation when carrying loads are consistently greater in males than females; however, when expressed relative to physical characteristics, these differences were minimal. Many studies included in the review utilised loaded marches of shorter duration, 5–10 min [42], at slower speeds (2.7–5.0 km/h) [43], using lighter loads (0–20 kg) than military contexts [44], often with civilians walking on treadmills. Considering this, studies of longer duration (90 min–3 h), with heavier loads (16–43 kg,) have shown an increased metabolic cost as measured by HR and VO_2max_ in females soldiers when compared to male soldiers [18,24]. In a similar manner to the results of this study, previous research in military training contexts has shown a significantly greater cardiovascular demand as measured by both average and peak heart rate in females performing load carriage tasks with loads as light as 15 kg or approximately 25% of their body weight [16]. The higher relative load carried by females (~9% more) likely contributed substantially to observed HR and VO_2_ differences. This scaling effect should be considered when interpreting sex-based comparisons.

All soldiers in this study, regardless of sex, were required to carry 30 kg of load, as in military contexts, the loads carried are dictated by the tasks and cannot be scaled to sex or body mass [41]. Given this, it is imperative that physical conditioning to carry load is undertaken [45]. In addition to carrying load every 10–14 days, previous research has highlighted the importance of both aerobic conditioning [32] and relative strength, particularly the upper limb [46] to load carriage performance. As an example, female soldiers in this study were working at a much higher relative intensity than the male soldiers with the average oxygen consumption by female soldiers during the load carriage task approximately 68% of their capacity as opposed to 46% for the male soldiers. Likewise, VO_2peak_ values by females were approximately 110% of their capacity, and 80% of the males. Although VO_2_peak values exceeded 100% of predicted VO_2_max (up to ~110%), this is not physiologically implausible. Predicted VO_2_max values were derived from 2.4 km run times and carry inherent error margins. Additionally, the steep incline during the march likely elicited near-maximal effort, similar to how heart rate can exceed age-predicted maximum under load carriage conditions. Therefore, these values should be interpreted as reflecting the high metabolic strain of the task rather than a measurement artefact. Despite the female soldiers’ predicted VO_2max_ of 45.0 mL/kg/min considered above average [47], any increases in aerobic fitness for female soldiers would enable them to work at a lower percentage of their capacity, and likely decrease injury risk [14]. Females also tend to have lower levels of upper limb strength than males, with the differences in lower limb strength being less pronounced [48]. Females therefore tend to make greater relative improvements in upper body strength than males with dedicated strength training due to this baseline difference [49].

Noting these differences in fitness levels, female soldiers exposure to military styled training tend towards greater improvements in physical fitness than males [50]. This greater increase is thought to be due to female soldiers entering training less physically fit than their training potential [51]. As such, optimised conditioning before training and once enlisted may help mitigate the load carriage metabolic gap between male and female soldiers [52]. While appropriate training may reduce this gap, some physiological difference may still exist, particularly under occupationally relevant loads [53].

This study is limited by a relatively small sample size, as is common in tactical populations; however, this is offset by the use of a linear mixed model which is a robust statistical analysis catering to small samples and repeated measures. Despite the small sample size, large effect sizes (β > 1 for HR) indicate meaningful differences that warrant further investigation in larger, more controlled studies. Despite these small numbers, this study is novel in that it collects field-based data of enlisted soldiers wearing occupational loads in a real-world context. Thus, the study provides insight into the physiological demands of formation load carriage, which can inform training and recovery practices for soldiers. Additional limitations include the inability to control for menstrual cycle phase, hydration status, variability in pack configuration and daily readiness factors (e.g., sleep quality, fatigue, nutrition), all of which may influence cardiovascular and metabolic responses and are relevant for sex-based physiological comparisons. These controlled variables may have introduced additional variability into the data and should be considered in future research designs.

## 5. Conclusions

Findings suggest females experience greater physiological strain under identical load carriage conditions, but conclusions should be considered exploratory given methodological constraints. Adequate recovery and pacing strategies should be used for these events. Optimising aerobic fitness and relative strength may contribute to reducing physiological strain. Given the greater relative loads carried by female soldiers in this study, lighter male soldiers might face similar challenges.

## Figures and Tables

**Table 1:** Demographic and baseline data.

Measure	Males (n = 6)	Females (n = 6)
Age (yrs)	26.3 ± 5.0	26.5 ± 6.6
Years of Experience (yrs)	7.2 ± 4.9	6.3 ± 5.5
Height (cm)	182.3 ± 6.2	167.4 ± 6.9 *
Uniformed weight (kg)	88.2 ± 8.7	70.9 ± 10.6 *
Relative load (%)	34.3 ± 3.4	43.2±7.5 *
Predicted VO_2_ (mL/kg/min)	56.7 ± 6.1	45.0 ± 2.9 *

* Significantly different from male soldiers *p* < 0.001.

**Table 2:** Fixed Effects Estimates of Heart Rate.

Term	b	SE b	df	t
Average HR				
Intercept	136.410	1.390	10	98.17
Sex (F)	−14.436	1.390	10	−10.389
Variant (B)	0.386	1.224	20	0.315
Variant (C)	1.419	1.224	20	1.159
Sex (F) × Variant (B)	1.569	1.224	20	1.282
Sex (F) × Variant (C)	−1.731	1.224	20	−1.413
Peak HR				
Intercept	168.917	1.324	10	127.571
Sex (F)	−11.25	1.324	10	−8.496
Variant (B)	1.417	0.899	20	1.577
Variant (C)	−0.583	0.899	20	−0.649
Sex (F) × Variant (B)	1.583	0.899	20	1.762
Sex (F) × Variant (C)	−2.25	0.899	20	−2.504

**Table 3:** Fixed Effects Estimates of VO_2_.

Term	B	SE B	df	t
Average VO_2_				
Intercept	26.100	1.49	4	17.521
Sex (F)	−4.678	1.49	4	−3.14
Variant (B)	0.033	0.657	8	0.051
Variant (C)	0.533	0.657	8	0.812
Sex (F) × Variant (B)	−0.922	0.657	8	−1.404
Sex (F) × Variant (C)	0.244	0.657	8	0.372
Peak VO_2_				
Intercept	45.639	1.72	4	26.561
Sex (F)	−3.972	1.718	4	−2.312
Variant (B)	0.644	0.619	8	1.04
Variant (C)	−1.089	0.619	8	−1.758
Sex (F) × Variant (B)	−0.011	0.619	8	−0.018

Intercept corresponds to the (unweighted) grand mean.

## Data Availability

The datasets analysed during the current study are not publicly available due to military data governance restrictions and participant confidentiality requirements. Data access is restricted under institutional and defence ethics approvals. Summary findings are presented in this article, but the raw data cannot be shared. Further information may be available from the corresponding author upon reasonable request and with appropriate institutional and defence permissions.

## Supplementary Materials

The following supporting information can be downloaded at: https://www.mdpi.com/article/10.3390/sports13120442/s1, Supplementary Table S1: Estimated marginal means for average and peak HR; Supplementary Table S2: Average marginal means for average and peak VO_2_.

## Abbreviations

The following abbreviations are used in this manuscript:

HR	Heart Rate
VO_2_	Oxygen Consumption
