# Supplementary material for: Sex Differences in the Metabolic Cost of a Military Load Carriage Task: A Field Based Study
Source: Sports (Basel). 2025 Dec 9;13(12):442. doi: 10.3390/sports13120442 (PMC12736546; doi:10.3390/sports13120442)
Supplement: Supplementary file 1 [file sports-13-00442-s001.zip › sports-4008998-supplementary-TableS1.pdf]

Supplementary Table S1: Estimated marginal means for average and peak HR.

				95% CI	
**Sex**	**Variant**	**Estimate**	**SE**	**Lower**	**Upper**
Average HR					
Male	A	123.933	2.619	118.800	129.067
Female	A	149.667	2.619	144.533	154.8
Male	B	121.667	2.619	116.533	126.8
Female	B	154.000	2.619	148.866	159.134
Male	C	120.333	2.619	115.2	125.467
Female	C	148.883	2.619	143.75	154.017
Peak HR					
Male	A	160.667	2.263	156.231	165.102
Female	A	180.000	2.263	175.565	184.435
Male	B	154.833	2.263	150.398	159.269
Female	B	181.833	2.263	177.398	186.269
Male	C	157.500	2.263	153.065	161.935
Female	C	178.667	2.263	174.231	183.102
