# Supplementary material for: Sex Differences in the Metabolic Cost of a Military Load Carriage Task: A Field Based Study
Source: Sports (Basel). 2025 Dec 9;13(12):442. doi: 10.3390/sports13120442 (PMC12736546; doi:10.3390/sports13120442)
Supplement: Supplementary file 1 [file sports-13-00442-s001.zip › sports-4008998-supplementary-TableS2.pdf]

Supplementary Table S2: Estimated marginal means for average and peak VO_2

Sex	Variant	Estimate	SE	95% CI Lower	95% CI Upper
Average VO_2					
Male	A	20.533	2.302	16.021	25.046
Female	A	31.733	2.302	27.221	36.246
Male	B	22.200	2.302	17.687	26.713
Female	B	31.067	2.302	26.554	35.579
Male	C	21.533	2.302	17.021	26.046
Female	C	29.533	2.302	25.021	34.046
Peak VO_2					
Male	A	42.300	2.583	37.237	47.363
Female	A	50.267	2.583	45.204	55.329
Male	B	39.633	2.583	34.571	44.696
Female	B	49.467	2.583	44.404	54.529
Male	C	43.067	2.583	38.004	48.129
Female	C	49.100	2.583	44.037	54.163
